# Paenarthrobacter sp. GOM3 Is a Novel Marine Species With Monoaromatic Degradation Relevance

**DOI:** 10.3389/fmicb.2021.713702

**Published:** 2021-08-03

**Authors:** Jaime Rosas-Díaz, Alejandra Escobar-Zepeda, Libertad Adaya, Jorge Rojas-Vargas, Diego Humberto Cuervo-Amaya, Ayixon Sánchez-Reyes, Liliana Pardo-López

**Affiliations:** ^1^Departamento de Microbiología Molecular, Instituto de Biotecnología, Universidad Nacional Autoónoma de México, Cuernavaca, Mexico; ^2^Unidad Universitaria de Secuenciación Masiva y Bioinformática, Instituto de Biotecnología, Universidad Nacional Autónoma de México, Cuernavaca, Mexico; ^3^Cátedras Conacyt – Instituto de Biotecnología, Universidad Nacional Autónoma de México, Cuernavaca, Mexico

**Keywords:** marine bacteria, aromatic compounds, hydrocarbon-degrading bacteria, Gulf of Mexico, bioprospection

## Abstract

Paenarthrobacter sp. GOM3, which is a strain that represents a new species-specific context within the genus *Paenarthrobacter*, is clearly a branched member independent of any group described thus far. This strain was recovered from marine sediments in the Gulf of Mexico, and despite being isolated from a consortium capable of growing with phenanthrene as a sole carbon source, this strain could not grow successfully in the presence of this substrate alone. We hypothesized that the GOM3 strain could participate in the assimilation of intermediate metabolites for the degradation of aromatic compounds. To date, there are no experimental reports of *Paenarthrobacter* species that degrade polycyclic aromatic hydrocarbons (PAHs) or their intermediate metabolites. In this work, we report genomic and experimental evidence of metabolic benzoate, gentisate, and protocatechuate degradation by *Paenarthrobacter* sp. GOM3. Gentisate was the preferred substrate with the highest volumetric consumption rate, and genomic analysis revealed that this strain possesses multiple gene copies for the specific transport of gentisate. Furthermore, upon analyzing the GOM3 genome, we found five different dioxygenases involved in the activation of aromatic compounds, suggesting its potential for complete remediation of PAH-contaminated sites in combination with strains capable of assimilating the upper PAH degradation pathway. Additionally, this strain was characterized experimentally for its pathogenic potential and *in silico* for its antimicrobial resistance. An overview of the potential ecological role of this strain in the context of other members of this taxonomic clade is also reported.

## Introduction

*Paenarthrobacter adv. Paene*, of which the translation from Latin is “almost” or “nearly” (Glare, 1968) (almost *Arthrobacter*), is a genus of gram-positive bacteria proposed recently from the reclassification of six species, namely, *Paenarthrobacter aurescens*, *Paenarthrobacter histidinolovorans*, *Paenarthrobacter ilicis*, *Paenarthrobacter nicotinovorans*, *Paenarthrobacter nitroguajacolicus*, and *Paenarthrobacter ureafaciens*, which belong to the *Arthrobacter* genus and, until now, have remained the only species with validated names (Busse, 2016). Most species of this genus have been isolated from soil, and they have a common peptidoglycan type A3α (Lys–Ala–Thr–Ala A11.17); a quinone system that contains menaquinone MK-9(H2); a polar lipid profile with the presence of diphosphatidylglycerol, phosphatidylglycerol, phosphatidylinositol, dimannosylglyceride, and monogalactosyldiacylglycerol; and a G+C content in genomic DNA from 61.3 to 63.6 (mol%) (Busse, 2016; Busse and Wieser, 2018).

Members of this genus have been reported to have interesting metabolic capabilities to degrade xenobiotics used in industrial applications and bioremediation. Examples include the degradation of the aromatic amine atrazine and similar compounds used as herbicides by *P. aurescens* TC1 (Strong et al., 2002), the utilization of nitroaromatic compounds by *P. nitroguajacolicus* (Kotoućková et al., 2004), nicotine metabolism by *P. nicotinovorans* (Baitsch et al., 2001), and nylon derivative degradation by *P. ureafaciens* KI72 (Takehara et al., 2017). Nevertheless, to date, there is no experimental evidence that strains of this genus are capable of degrading aromatic compounds, such as polycyclic aromatic hydrocarbons (PAHs) or their intermediate metabolites.

Aromatic compounds can be defined as organic molecules formed by one or more aromatic rings. The shape of the ring provides structural and chemical stability due to a symmetric system of π electrons, which makes them recalcitrant compounds in the environment (Vogt et al., 2011). These aromatic compounds are found in nature as lignin, amino acids and tannins, although other sources also include anthropogenic activities, such as agricultural (herbicides and insecticides), industrial (solvents, detergents and petroleum), and domestic sources (Cinar, 2004). Many of these compounds that are released to the environment are principal environmental pollutants due to their toxicity to living beings (Cao et al., 2009). In the environment, these compounds can be subjected to diverse physical, chemical, and biological phenomena that lead to their transformation, elimination or transport to other environmental compartments through evaporation, dilution, precipitation, lixiviation, sequestration, abiotic reactions (hydrolysis, photooxidation, or chemical oxidation), bioaccumulation, or microbial biodegradation (Hassanshahian et al., 2015).

The aerobic bacterial biodegradation of aromatic compounds has been divided into upper pathways, which begin from the transformation of the original compound into a few central intermediates (catechol, gentisate, and protocatechuate), and lower pathways, in which the aromatic ring of the intermediates is cleaved, producing into intermediary metabolites (acetyl-CoA, succinyl-CoA, and pyruvate) (Fuchs et al., 2011). However, it is well-documented that in some cases, during the degradation of compounds, such as PAHs, where there are multiple upper pathways involved, it is possible to detect intermediate metabolites as a result of incomplete biodegradation. This suggests that biodegradation occurs mainly in several upper pathways and only in some lower pathways (Luan et al., 2006) and that the production of intermediate metabolites is a common phenomenon observed in the partial degradation of PAHs (Cinar, 2004; Dastgheib et al., 2012).

The superior routes of degradation consist of one oxidation catalyzed by nonheme Rieske iron oxygenases, flavoproteins and soluble di-Fe monooxygenases, which are responsible for the activation and subsequent degradation of the aromatic ring (Gibson and Parales, 2000) and are classified as monooxygenases (hydroxylases) or dioxygenases (Parales and Resnick, 2004; Huijbers et al., 2014). Monooxygenases catalyze the cleavage of the oxygen-oxygen bond of O_2_, inserting an oxygen atom within the aromatic ring and forming phenols and later catechols (Ladino-Orjuela et al., 2016). Dioxygenases, on the other hand, carry out the dihydroxylation of the aromatic ring, resulting in the formation of a cis-dihydrodiol, which is rearomatized toward an intermediate diol by the action of a dehydrogenase (Mallick et al., 2011). The transformation of these structurally diverse aromatic compounds through peripheral metabolic pathways yields key intermediates, such as catechol (1,2-dihydroxybenzene), protocatechuate (3,4-dihydroxybenzoate), and gentisate (2,5-dihydroxybenzoate), which are subsequently channeled through a few central (lower) pathways into cellular metabolism (Cao et al., 2009). Once in the lower pathways, these catabolites are metabolized by intra- or extradiol dioxygenase enzymes, each of which can cleave the aromatic ring *via* the intradiol (ortho) and extradiol (meta) routes, respectively (Fritsche and Hofrichter, 2008). The intradiol reaction that takes place between the two hydroxyl groups is carried out by a dioxygenase using Fe(II) as a cofactor (Guzik et al., 2013). The extradiol reaction that occurs at the carbon-carbon bond adjacent to one of the hydroxyl groups is carried out by dioxygenases using Fe(III) as a cofactor (Suenaga et al., 2014).

Despite the many reports about aromatic compound degradation by bacteria, from PAHs to central intermediates, there is no study that evaluates the bacterial degradation capability of mixtures of central intermediates (benzoate, gentisate, and protocatechuate), which is how aromatic compounds are found in nature (Landry and Tremblay, 2012).

In the present study, we report the isolation of a bacterium from the southwestern Gulf of Mexico named GOM3, which, according to genomics and experimental results, is capable of degrading central intermediates related to the lower degradation pathway of aromatic compounds. The ecological role of this strain and a first approach to its pathogenicity have also been reported. To date, taxonomic analyses suggest that this strain represents a novel genomospecies of the genus *Paenarthrobacter*, which would make it the first species of this genus isolated from marine sediments.

## Materials and Methods

### Sample Collection and Strain Isolation

To obtain bacterial isolates, 1 g of marine sediment from station S03 collected in June 2015 (18°44′24.0′′N 94°30′00.0′′W, 275 m depth) was added to 50 mL of minimum medium composed of the following compounds (g⋅L^–1^): Na_2_SO_4_, 0.183; CaCl_2_, 0.073; NH_4_Cl, 0.267; MgCl_2_⋅6H_2_O, 0.16; NaMoO_4_⋅2H_2_O, 0.0002; FeSO_4_, 0.005; NaCl, 11.68; K_2_HPO_4_, 0.8; KH_2_PO_4_, 0.2; and phenanthrene, 0.01% w/v, as the sole carbon source. The cultures were incubated at 200 rpm and 30°C for 3 months, and every 30 days, the cultures were transferred to fresh minimum medium.

For the isolation of strains from consortia enriched with phenanthrene as the sole carbon source, serial dilutions were prepared up to 10^–6^, and 100 μL of the strain was plated in solid EDM containing the following compounds (g⋅L^–1^): NaCl, 23.6; KCl, 0.64; MgCl_2_⋅6H_2_O, 4.53; MgSO_4_⋅7H_2_O, 5.94; CaCl_2_⋅2H_2_O, 0.98; agar, 15; tryptone, 5; and yeast extract, 2.5. The Petri dishes were incubated for 7 days at 30°C. The strains were cultured in minimal medium (described previously) with 0.01% phenanthrene (w/v), and only strain GOM3 showed slight growth.

### Nucleic Acid Extraction and Genome Sequencing

An isolated colony from strain GOM3 was grown overnight at 30°C and 180 rpm in 25 mL of LB medium in a 125 mL flask, and total DNA was extracted using a Quick-DNA^TM^ Miniprep Kit from Zymo Research (Irvine, CA, United States) following the kit instructions. Sequencing was performed on an Illumina NextSeq 500 platform following a paired-end protocol of 75 cycles (San Diego, CA, United States) at the Unidad Universitaria de Secuenciación Masiva y Bioinformática, UNAM, México. Polymerase chain reaction (PCR) was performed using Taq polymerase with the BAC primers F27 and R1492 for 16S rDNA (Heuer et al., 1997 referenced by Monciardini et al., 2002). The PCR product was purified with DNA Clean & Concentrator-25 from Zymo Research and subsequently sequenced by Sanger in the DNA Synthesis and Sequencing Unit of the Institute of Biotechnology, UNAM, México.

### Genome Assembly and Refinement

After adapter sequence trimming and control quality filtering, the paired-end reads obtained from Illumina sequencing were used for *de novo* genome assembly with Velvet v1.2.10 (Zerbino, 2010), with a k-mer size of 63. To improve the quality of the assembly, we used REAPR v1.0.18 (Hunt et al., 2013) for correcting misassembled scaffolds, BESST v2.2.5 (Sahlin et al., 2014) for scaffolding, GapFiller v1-10 (Nadalin et al., 2012) for filling gaps between scaffolds and iCORN2 v0.95 (Otto et al., 2010) for correcting errors in scaffolds. Contamination and the completeness of the final version of the assembly were evaluated using the CheckM v1.0.12 toolkit (Parks et al., 2015).

### Taxonomic Identification and Phylogenetic Analysis

For the first taxonomic identification, the 16S gene sequence obtained by Sanger was assembled by CAP in BioEdit (Hall, 1999) and annotated on Blast (McGinnis and Madden, 2004) using the 16S ribosomal RNA sequences for the Bacteria and Archaea database (consulted on June 03, 2021). To obtain phylogenetic neighbors with standing in nomenclature, the genome of *Paenarthrobacter* sp. strain GOM3 was compared against a custom database containing all type strain genomes available in the NCBI assembly portal (https://www.ncbi.nlm.nih.gov/assembly, consulted on April 01, 2021) (Sánchez-Reyes and Fernández-López, 2021) *via* the MASH algorithm (Ondov et al., 2016). The twenty types strains with the smallest MASH distances were chosen for further analysis *via* average nucleotide identity (ANI) determination with fastANI (Jain et al., 2018), digital DNA-DNA hybridization (dDDH) (Meier-Kolthoff et al., 2013) and a phylogenomic reconstruction with a set of 92 bacterial core genes included in UBCG software (Na et al., 2018). For the phylogenomic reconstruction, we also added 27 RefSeq genomes for the *Paenarthrobacter* lineage on NCBI (consulted on April 29, 2021). The final version of the genome assembly was used for taxonomic labeling against the Genome Taxonomy Database using GTDBtk v1.3.0 (Chaumeil et al., 2019).

### Inference of Putative Protein Functions and Pangenomics Analysis

Functional annotation was performed on CDSs predicted from contig sequences using GeneMark V4.32 (Besemer et al., 2001). Genes translated to proteins were submitted to the RAST server for functional inference (Aziz et al., 2008). This annotation was complemented by the following prediction of specific genomic features: (1) antimicrobial resistance gene prediction using the Resistance Genes Identifier (RGI) pipeline v5.1.1 with the CARD database v3.1.0. (Alcock et al., 2019), (2) analysis of secondary metabolite biosynthetic gene clusters using antiSMASH v5.1.2 (Blin et al., 2019) with the option of “relaxed” strictness, and 3) PATRIC v3.6.9 annotation for virulence factor identification (Wattam et al., 2014).

Twelve genomes annotated as *Paenarthrobacter* species were representatives in the GTDB database (NCBI accession IDs: *P. sp000526335* GCF_000526335, *P. sp002979775* GCF_002979775, *P. sp001512285* GCF_001512285, *P. sp006964045* GCF_006964045, *P. sp000281065* GCF_000281065, *P. aurescens_A* GCF_000014925, *P. aurescens* GCF_006538985, *P. nicotinovorans_A* GCF_000514015, *P. sp002224285* GCF_002224285, *P. sp900106835* GCF_900106835, *P. sp001423565* GCF_001423565, and *P. ureafaciens* GCF_004028095). An assembly of *Paenarthrobacter* sp. *HW13* (IMG/ER study ID: Gs0118559), which is a strain reported to harbor aromatic degradation genes (Moraes et al., 2018), was also used, and the closest strains according to GBDP distances (NCBI accession IDs: *P. aurescens NBRC12136* GCA_006538985, *P. ureafaciens DSM20126* GCA_004028095) were used to conduct pangenomics analysis using the Panaroo tool v1.2.4 (Tonkin-Hill et al., 2020) with the following parameters: –clean-mode strict; –alignment core; –core_threshold 1; –refind_prop_match 0.75; and –merge_paralogs. The aim of this analysis was to determine the uniqueness of this new species in terms of gene diversity and its functions in the context of close reference genomes. Sequences of proteins detected as unique to the strain of interest were mapped within the Kyoto Encyclopedia of Genes and Genomes (KEGG) database through their Automatic Annotation Server (KAAS v2.1) using the single-directional best hit (SBH) method (Moriya et al., 2007). Additionally, an enrichment of metabolic pathways in biochemical cycle analysis was performed using all the amino acid sequences in Multigenomic Entropy Based Score (MEBS) software (De Anda et al., 2017), with a restrictive false discovery rate (FDR) of 0.0001.

### Experimental Evidence of Aromatic Compound Degradation

An overnight culture of GOM3 was grown in LB medium at 30°C and 180 rpm. A beginning aliquot was calculated to result in an OD600 nm near 0.1 in 5 mL of fresh minimum medium with benzoate, gentisate or protocatechuate individually at a concentration of 0.1%. When the mixtures of substrates were used, we adjusted each compound to 0.05% in pairwise mixtures and to 0.033% when the mixture contained all three compounds (concentrations are expressed as % w/v). Due to the acidic nature of these compounds, they were previously neutralized with 5 M NaOH to pH 7, and all media were passed through 0.45 μm filters. The experiments were carried out in biological triplicates. The aromatic compound concentration was measured with two different strategies. In the presence of one or two aromatic compounds, we used UV microplates in a Synergy 2 Multi-Mode de BioTek microplate lector. Benzoate, gentisate, and protocatechuate were measured at 230 nm (Cabel et al., 2000), 330 and 260 nm (Robbins, 2003), respectively. Due to absorbance interference from mixed substrates, when the compounds were in a mixture, we used an Agilent 1220 high-performance liquid chromatography model equipped with a Phenomenex Luna 5 μm C18 column (100 Å 150 mm × 3 mm). We used a gradient method for separating each compound, and the absorbance was read at the same wavelength described previously. To calculate the concentration, we employed a calibration curve.

The specific growth rate (μ) and duplication time were estimated when strain GOM3 was in an exponential growth phase. Both kinetic parameters were estimated using equations described by Widdel (2007). For the specific growth rate, we used the following equation:

μ=(lnOD-2lnOD)1/(t-2t)1

where μ is the specific growth rate, OD is the optical density, and *t* is the time.

To calculate the duplication time, we used the following equation:

t=dln2/μ

where μ is the specific growth rate and t_*d*_ is the duplication time.

Finally, to measure the volumetric consumption rate, we plotted time against the OD and computed the line slope when the compound concentration decreased.

### Pathogenicity Assessment

The strain was grown in minimum medium with 3 g⋅L^–1^ peptone and 3 g⋅L^–1^ yeast extract for 20 h at 30°C. The cells were harvested by centrifugation, washed three times with 10 mM sterile MgSO_4_ and resuspended in sterile phosphate buffered saline (PBS). *Galleria mellonella* was used as a pathogenicity model for pathogenicity assays (Cools et al., 2019). To determine the health index (HI) (Loh et al., 2013), a group of 10 larvae was injected in triplicate with 100 CFU/10 μL following the protocol reported by Cools et al. (2019) and incubated at 30°C in the dark. The larvae were monitored for 5 days. One hundred CFU/10 μL *Pseudomonas aeruginosa* ATCC 27853 strain and 100 CFU/10 μL *Escherichia coli* DH5α were used as positive and negative controls, respectively. To calculate the median lethal dose (LD_50_), triplicate larval groups (*n* = 15) were injected with 10^2^, 10^4^, 10^6^, 10^8^, and 10^10^ CFU/10 μL strain. The larvae were incubated at 30°C and scored as dead or alive 48 h post-infection. Larvae were considered dead if they displayed no movement even with stimulation. The LD_50_ was calculated using a probit regression model with the “ecotox” library in R (Hlina et al., 2019).

## Results

### Genome Sequencing and Bioinformatic Analysis

The refined version of the genomic assembly had a 63.2% GC content and a length of 4.4 Mbp encompassed in 50 fragments. Nonetheless, the N50 value was 2.3 Mbp, and the L50 was seven contigs. This assembly was nearly complete (99.71%) and contained a low proportion of contaminating sequences, representing 0.39% of the assembly according to the CheckM tool. In total, 4,140 genes were predicted. A complete operon of rRNA containing 16S, 5S, and 23S was found, and a total collection of 52 genes for tRNAs was identified. This Whole Genome Shotgun project has been deposited at DDBJ/ENA/GenBank under the accession JADIXW000000000. The version described in this paper is version JADIXW010000000.

### Taxonomic Identification and Intergenomic Distance Inference Through ANI

The 16S rDNA sequence of the GOM3 strain had the highest identity (95.07%) with *P. nitroguajacolicus* strain G2-1 16S (accession number NR_027199.1), with a query coverage of 98%. Whole-genome similarity metrics allowed us to assess the taxonomic circumscription for strain-allowed *Paenarthrobacter*, *Pseudoarthrobacter*, or *Arthobacter* genomes, suggesting a continuum of diversity among this strain and its closest relatives (Table 1). Additionally, the estimation of dDDH notably indicated the absence of a species-specific relationship within the *Paenarthrobacter*/*Arthrobacter/Pseudoarthrobacter* clades. Therefore, we concluded that *Paenarthrobacter* sp. GOM3 is a novel species within the *Paenarthrobacter* genus (*Micrococcaceae* family).

**TABLE 1:** Pairwise comparisons of *Paenarthrobacter* sp. GOM3 against closets type strain genomes according to several overall genome relatedness indexes (Mash D, ANI, and digital DNA-DNA hybridization (dDDH).

RefSeq assembly accession	Reference organism	ANI (%)	Mash D	Matching-hashes	dDDH (%)
GCF_014647595.1	*Paenarthrobacter histidinolovorans* JCM 2520	82.58	0.135	30/1000	52.90
GCF_014648735.1	*Paenarthrobacter nicotinovorans* JCM 3874	82.52	0.135	30/1000	54.30
GCF_006538985.1	*Paenarthrobacter aurescens* NBRC 12136	82.10	0.130	34/1000	53.90
GCF_016907545.1	*Paenarthrobacter ilicis* DSM 20138	81.78	0.142	26/1000	51.70
GCF_004028095.1	*Paenarthrobacter ureafaciens* DSM 20126	79.91	0.168	15/1000	35.50
GCF_013409905.1	*Arthrobacter cupressi* DSM 24664	77.82	0.174	13/1000	24.00
GCF_003369445.1	*Arthrobacter silvisoli* NEAU-SA1	77.78	0.182	11/1000	22.60
GCF_000238915.1	*Arthrobacter globiformis* NBRC 12137	77.01	0.197	8/1000	19.00
GCF_004354015.1	*Arthrobacter terricola* JH1-1	76.78	0.211	6/1000	19.80
GCF_017052465.1	*Arthrobacter pascens* DSM 20545	76.66	0.197	8/1000	19.00
GCF_001046895.1	*Pseudarthrobacter siccitolerans* 4J27	76.57	0.211	6/1000	18.80
GCF_003634095.1	*Arthrobacter oryzae* DSM 25586	76.55	0.204	7/1000	17.40
GCF_014644495.1	*Pseudarthrobacter polychromogenes* CGMCC 1.1927	76.54	0.204	7/1000	19.00
GCF_014639275.1	*Arthrobacter liuii* CGMCC 1.12778	76.49	0.192	9/1000	18.00
GCF_014712225.1	*Pseudarthrobacter sulfonivorans* ALL	76.44	0.204	7/1000	18.20
GCF_001457025.1	*Pseudarthrobacter enclensis* NIO-1008	76.43	0.197	8/1000	19.80
GCF_014644515.1	*Pseudarthrobacter scleromae* CGMCC 1.3601	76.37	0.211	6/1000	18.90
GCF_900105535.1	*Pseudarthrobacter equi* IMMIB L-1606	76.36	0.192	9/1000	19.40
GCF_000022025.1	*Pseudarthrobacter chlorophenolicus* A6	76.34	0.182	11/1000	18.40
GCF_011927905.1	*Arthrobacter pigmenti* DSM 16403	71.27	0.220	5/1000	13.60

*The table is ordered by column ANI in descending order.*

**FIGURE 1:**
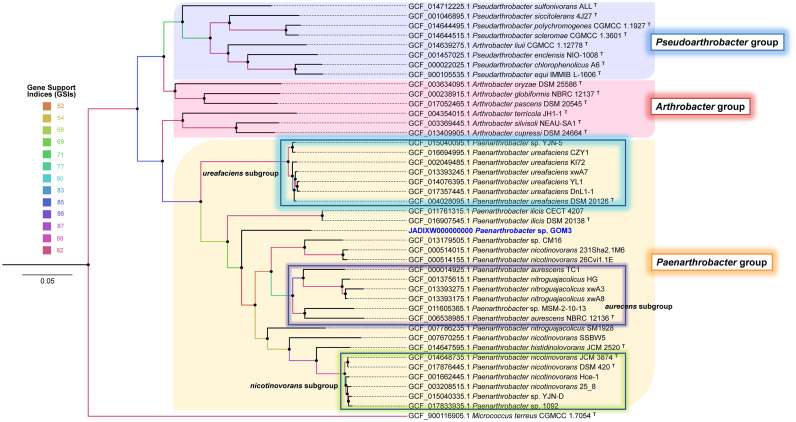
Phylogenomic unrooted tree inferred with 92 housekeeping bacterial core genes in the UBCG pipeline. Gene support indices (GSIs) are represented in color scale per node. Bar 0.05 substitution per site. The *Micrococcus terreus* CGMCC 1.7054^*T*^ genome was used as an outgroup.

A phylogenomic approach using 92 single-copy genes extracted from query and neighboring genomes with UBCG software also confirmed that *Paenarthrobacter* sp. GOM3 is a novel species identified by clustering as an independent branch within the *Paenarthrobacter-*type species clade (Figure 1) close to *Paenarthrobacter ilicis* DSM 20138. The coherent separation of the *Paenarthrobacter/Arthrobacter/Pseudoarthrobacter* groups into three independent clades is highly supported by the gene support indices (GSIs).

### Ecological Role of *Paenarthrobacter* sp. GOM3

According to the Genome Taxonomy DataBase (GTDB), our assembly is classified at the genus level as *Paenarthrobacter*. The closest relative to our strain is *Paenarthrobacter* sp. 001423565, which was isolated from an Arabidopsis leaf (NCBI:txid1736274). Twelve representative genomes belonging to *Paenarthrobacter* species were used for pangenomic analysis. The assemblies of *P. aurescens* NCBR 12136, *P. ureafaciens* DSM 20126 and *Paenarthrobacter* sp. *strain* HW13 were also included.

We observed a core of genes comprising 2,046 sequences out of the 13,402 sequences forming the pangenome. *Paenarthrobacter* sp. GOM3 has 565 unique genes, most of which have unknown functions (71.3%) according to PROKKA annotation. Nonetheless, relevant potential functions were found in these unique genes when mapping vs. the KEGG database, such as catechol and benzoate/toluate dioxygenases for aromatic compound degradation, and a collection of permeases for a variety of sugars and other substrates, such as shikimate and glycerol, and ions, such as nickel and cyanate (Supplementary Table 1).

To better understand the ecological role of this strain, the mechanisms of biochemical pathways involving S, C, O, Fe, and N were evaluated based on amino acid sequences for each strain in the pangenomic analysis. This analysis revealed that the highest score for our strain corresponded to the nitrogen cycle (Pfam entropy score 12.302), indicating that these bacteria could use nitrogen compounds, such as ammonia or nitrates, as energy sources. The second highest score corresponded to oxygen (score 4.493), agreeing with the aerobic nature of the strain, and the third corresponded to carbon (score 1.241), suggesting that this strain can degrade methane methyl compounds, such as methylamine, a common compound in the marine ecosystem. A negative score for sulfur (score -0.644) indicated that this *Paenarthrobacter* did not have all the pathways of sulfur cycle.

**FIGURE 2:**
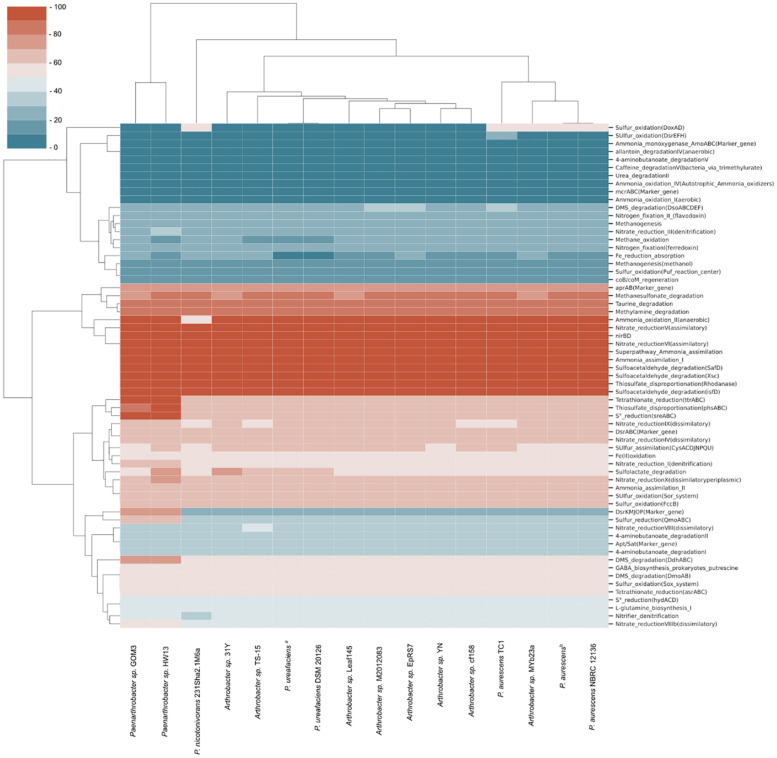
Metabolic completeness of C, O, N, S, and Fe cycles of strains related to pangenomic analysis. The most complete pathways in biogeochemical cycles are marked in red, and the least complete pathways shift to blue. *P. ureafaciens*^*a*^ corresponds to RefSeq assembly accession GCF_006538985, and *P. aurescens*^*b*^ corresponds to RefSeq assembly accession GCF_004028095.

Comparing our strain with the 16 strains from the pangenomic group, a heatmap was generated to identify the completeness of specific pathways in the reference genomes, revealing potential metabolic feature completeness (Figure 2). Ten metabolic pathways with 100% completeness were present in almost all the samples, including some reactions of the nitrogen cycle, such as ammonia assimilation (I), nitrate reduction (V and VI), superpathway ammonia assimilation and nitrate reductase (*nirBD*) enzyme. Other pathways with 100% completeness included sulfoacetaldehyde degradation and the widespread rhodanase enzyme involved in detoxification of cyanide. There was a reduced number of genes involved in methane compounds or nitrogen fixation. Pfam domains involved in tetrathionate S_4_O_6_^–2^ (*ttrABC*) and S^0^ (*sreABC*) reduction were only present in two strains, *Paenarthrobacter* sp. GOM3 and *Paenarthrobacter* sp. HW13, with 100% completeness, indicating that they likely use tetrathionate and elemental sulfur as a source of energy and electron acceptors. We could not identify the key enzyme marker for methane metabolism in any of the strains, e.g., the methyl-coenzyme M reductase (MCR) complex.

### Functional Annotation Analysis and Experimental Support

Genome annotation of *Paenarthrobacter* sp. GOM3 showed genes related to the degradation of aromatic compounds, such as benzoate, gentisate, and protocatechuate (Supplementary Tables 2–4, respectively).

**FIGURE 3:**
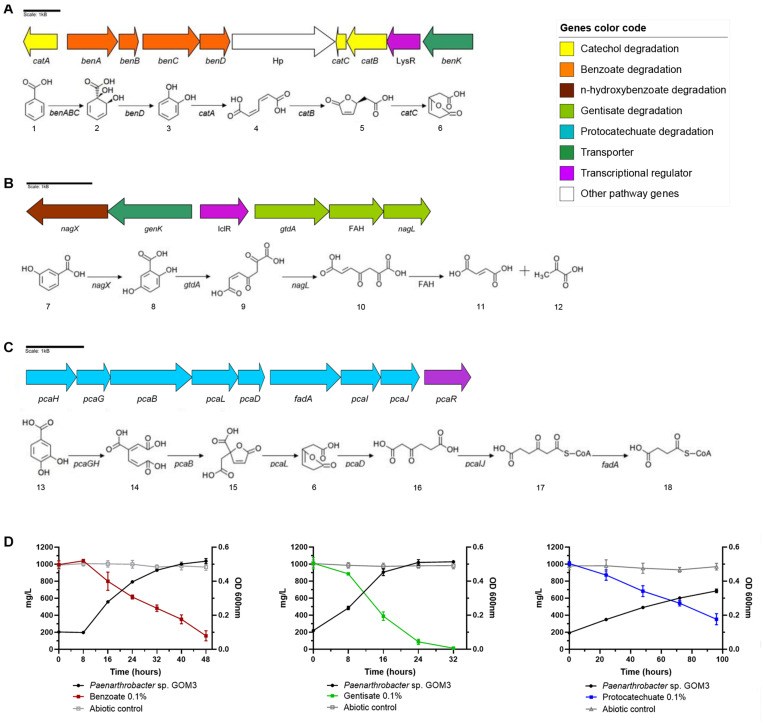
Genomic and experimental evidence of aromatic compound degradation by *Paenarthrobacter* sp. GOM3. Degradation pathways and genomic context are shown for three aromatic compounds: **(A)** benzoate, **(B)** gentisate, and **(C)** protocatechuate. **(D)** Growth kinetics and decrease in its corresponding compound concentration. Whiskers represent the standard deviation of three biological replicates. Metabolic compounds: (1) benzoate; (2) cis-1,2-dihydroxycyclohexa-3,5-diene-1-carboxylate; (3) catechol; (4) cis,cis-muconate; (5) (+)-muconolactone; (6) 3-oxoadipate enol-lactone; (7) 3-hydroxybenzoate; (8) gentisate; (9) 3-maleylpyruvate; (10) 3-fumarylpyruvate; (11) fumarate; (12) pyruvate; (13) protocatechuate; (14) beta-carboxy-cis,cis-muconate; (15) gamma-carboxymuconolactone; (16) 3-oxoadipate; (17) 3-oxoadipyl-CoA; and (18) succinyl-CoA. Concentrations of each aromatic compound in abiotic controls in gray.

In the case of benzoate, this strain has catabolic *benABCD* genes that convert benzoate into catechol (Carrington et al., 1994) and *catBCA* genes that transform catechol into 3-oxoadipate-enol-lactone (Houghton et al., 1995). In addition, GOM3 possesses a *benK* gene from the major facilitator superfamily (MFS) reported as a transporter of benzoate into the cell (Collier et al., 1997). Additionally, in this genomic context, a transcriptional regulator belonging to the LysR family was found (Maddocks and Oyston, 2008; Figure 3A). To verify the capacity of GOM3 to use the aromatic metabolic pathways found in its genome, this strain was grown in the presence of benzoate as the only carbon source, demonstrating that these genes are functional. Strain GOM3 had a specific growth rate (μ) of 0.087 ± 0.002 h^–1^ and a volumetric consumption rate of 0.027 ± 0.002 g/L h^–1^ (Supplementary Table 5), although it had a *lag* phase of 8 h in which it did not grow and the benzoate concentration did not decrease (Figure 3D, see benzoate growth kinetics and substrate degradation).

Two catabolic genes related to the gentisate degradation pathway were found in the genome—gentisate 1,2 dioxygenase *gtdA* and maleylpyruvate isomerase *nagL* (Zhou et al., 2001)—and in the vicinity of these genes, we identified a transporter *genK* (Xu et al., 2012), plus three more copies in the genome and a transcriptional regulator that belongs to the isocitrate lyase regulator type (IclR type) (Molina-Henares et al., 2006). Moreover, we found the *nagX* gene, which encodes a putative n-hydroxybenzoate hydroxylase that converts 3-hydroxybenzoate into gentisate (Park et al., 2007), and a gene that encodes a protein of the fumarylacetoacetate hydrolase family (Figure 3B). The μ of this strain was similar to that observed for the benzoate substrate (0.089 ± 0.003 h^–1^); however, it did not show a *lag* phase in the presence of gentisate (see Figure 3D, gentisate growth kinetics and substrate degradation). The volumetric consumption rate of gentisate was 0.039 ± 0.002 g/L h^–1^ (Supplementary Table 5).

Genes related to protocatechuate catabolism were also identified in the GOM3 genome responsible for converting this aromatic compound into the intermediary metabolite succinyl-CoA (Kowalchuk et al., 1994; Figure 3C). In the same genomic context, we found the transcriptional regulator *pcaR* (Romero-Steiner et al., 1994). In comparison with the other two substrates, the growth phase was more prolonged (see Figure 3D, protocatechuate growth kinetics and substrate degradation), and the μ and volumetric rate consumption values were lower, 0.02 ± 0.002 h^–1^ and 0.007 ± 0.001 g/L h^–1^, respectively (Supplementary Table 5).

**FIGURE 4:**
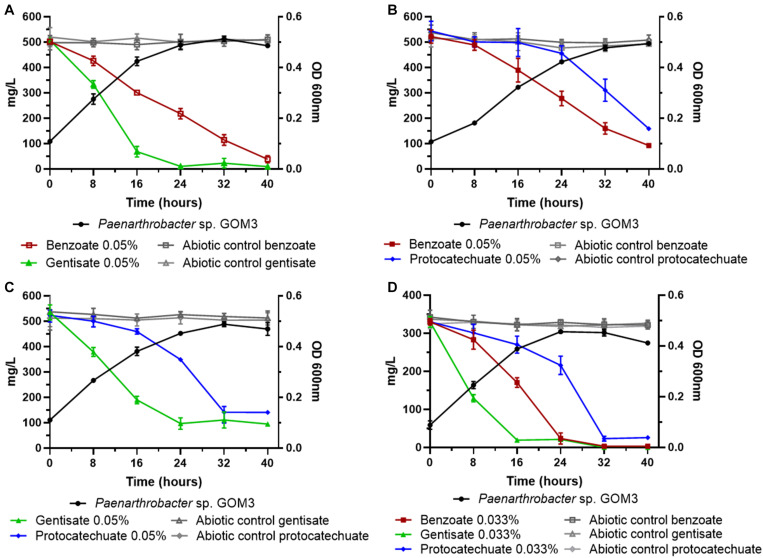
Substrate decrease and growth kinetics of *Paenarthrobacter* sp. GOM3 in the presence of different mixtures of aromatic compounds **(A)** benzoate and gentisate, **(B)** benzoate and protocatechuate, **(C)** gentisate and protocatechuate and **(D)** the three compounds. Whiskers represent the standard deviation of three biological replicates. Aromatic compound in abiotic controls in gray.

In addition, we tested the degradation capacity of GOM3 in mixtures of aromatic compounds. We observed that benzoate and gentisate (B/G) mix substrates were degraded simultaneously (Figure 4A), but the volumetric consumption rate of gentisate (0.028 ± 0.002 g/L h^–1^) was greater than that of benzoate (0.013 ± 0.001 g/L h^–1^), with a Wilcoxon test *P*-value of 0.05. The degradation of both substrates was complete after 40 h (Figure 4A). The specific growth rate in this mix was 0.085 ± 0.003 h^–1^.

In the mixture of benzoate and protocatechuate (B/P), we observed that the benzoate concentration decreased after 16 h, while the protocatechuate concentration started decreasing after 24 h (Figure 4B); however, both substrates had similar volumetric consumption rates (Wilcoxon test *P* = 0.13) (Supplementary Table 6), and the μ for this mix was 0.069 ± 0.002 h^–1^.

When gentisate and protocatechuate were mixed (G/P), the volumetric consumption rate of gentisate was higher than that of protocatechuate (Wilcoxon test *P* = 0.05) (Figure 4C and Supplementary Table 6), and the μ value of the mix was 0.077 ± 0.003 h^–1^.

Finally, when all three aromatic compounds were present, we observed that all substrate concentrations decreased simultaneously (Figure 4D), but at different rates. The compound with the highest volumetric consumption rate was gentisate (0.019 ± 0.001 g/L h^–1^), followed by benzoate (0.013 ± 0.001 g/L h^–1^) and protocatechuate (0.009 g/L h^–1^) (global Wilcoxon test *P* = 0.027).

### Pathogenicity Assessment

Some strains of the *Arthrobacter* genus are reported as opportunistic human pathogens (Huang et al., 2005). These strains are associated with clinical features, such as bacteremia, periodontitis, and endocarditis (Bernasconi et al., 2004). Because our strain is related to this genus (Figure 1), a first glimpse of its pathogenicity is shown below. An HI assay with 100 CFU/10 μL inoculum injected into *G. mellonella* showed that *Paenarthrobacter* sp. GOM3 was able to cause infection at a lower level than the pathogen *P. aeruginosa* ATCC 27853, in contrast with the negative controls (Supplementary Figure 1A). Using the doses described in the section “Materials and Methods,” a dose-response curve at 48 h was obtained (Supplementary Figure 1A). With 10^6^ CFU, nearly 40% of the larval population was dead, 90% died with 10^8^ CFU exposure, and 100% died with 10^10^ CFU exposure. The LD_50_ calculated for our strain was 2.29 × 10^6^ CFU at 48 h, with a confidence level of 0.95. In contrast with the *P. aeruginosa* LD_50_ of 5–17 CFU between 24 and 48 h (Miyata et al., 2003; Sonnleitner et al., 2003; Andrejko et al., 2014), the *E. coli* LD_50_ between 1 and 5 × 10^4^ CFU at 120 h (Alghoribi et al., 2014), and the *Klebsiella pneumoniae* LD_50_ of 1 × 10^4^ CFU at 72 h (Insua et al., 2013), the LD_50_ value of our strain is higher, indicating that the GOM3 strain is less harmful than recognized pathogens.

This sign of stress is related to possible pathogenic mechanisms of our strain and the presence of some virulence factors in its genome, according to PATRIC annotation, such as the *ilvD* (78% amino acid identity) and *icl* (79% identity) genes. These two genes do not have an obvious association with virulence mechanisms; however, experimental evidence supports that they are linked with virulence attenuation in *Mycobacterium tuberculosis* when the gene is inactivated, which makes them a good target for drug and vaccine development (Muñoz-Elías and McKinney, 2005; Singh et al., 2011). Additionally, it is suggested that our strain may have other virulence factors, with identities of approximately 60%, such as *cspA* (68%) for survival, *groL1* (60%) for adherence, *TetR (59%)* for regulation, and *ideR* (57%) for iron uptake.

In addition, we performed an *in silico* screening for genes related to antimicrobial resistance to assess the potential of GOM3 to succeed in the presence of this kind of drug. Two antimicrobial resistance genes were found in the *Paenarthrobacter* sp. GOM3 genome using the RGI pipeline. One gene was annotated as an ATP-binding cassette ribosomal protection protein capable of providing protection to a variety of drug classes, such as macrolide, lincosamide, streptogramin, tetracycline, oxazolidinone, phenicol, and pleuromutilin antibiotics. The other gene was annotated as a resistance-nodulation-cell division (RND) antibiotic efflux pump, with potential function against macrolide, monobactam, tetracycline, and aminocoumarin antibiotics. Further inspection of PROKKA functional annotation yielded a list of the following genes associated with antimicrobial resistance: 11 copies of fosfomycin resistance protein AbaF (*abaF*); 3 copies of linearmycin resistance ATP-binding protein LnrL (*lnrL*); 2 copies of putative multidrug resistance protein MdtD (*mdtD*), multidrug resistance protein Stp (*stp*), and multidrug resistance protein 3 (*bmr3*); and one copy of tetracycline resistance protein class C (*tetA*), antiseptic resistance protein (*qacA*), multidrug resistance protein NorM (*norM*), multidrug resistance protein MdtL (*mdtL*), putative multidrug resistance protein EmrY (*emrY*), daunorubicin/doxorubicin resistance ATP-binding protein DrrA (*drrA*), and bicyclomycin resistance protein (*bcr*).

## Discussion

The GOM3 strain represents a new species-specific context within the *Paenarthrobacter* genus (for which there are only six species described) and is grouped into an independent branch, with early divergence within it. In addition to phylogenetics, several lines of genomic evidence support this observation, highlighting the low nucleotide-level genomic similarities (ANI < 95% dDDH < 70%), which constitute standards in current microbial classification schemes, especially in the definition of genomospecies (Figure 1 and Table 1). The phylogenetically closest strains are *P. ilicis* DSM 20138, *Paenarthrobacter* sp. CM16, and *P. nicotinovorans* strains 231Sha2.1M6 and 26Cvi1.1E, and the latter two strains were probably misclassified at the species level since they clustered outside the nicotinovorans subgroup (see the highlighted green square in Figure 1). Although the taxonomic description of the GOM3 strain is beyond the scope of this paper, our phylogenomic reconstruction supports that the *Paenarthrobacter* clade is monophyletic and that it has undergone significant radiation with coherently defined genomic groups (*P. urefaciens*, *P. aurecens*, *P. nicotinovorans* subgroups, and the species *P. ilicis* and *P. histidinolovorans* were less populated at the genomic level but well-separated from the other groups). The GOM3 strain is clearly an independently branched member of any group described thus far. Although the CM16 strain was isolated from a halophytic endophytome, specifically from roots of the coastal plant *Cakile maritima*, the GOM3 strain of this study represents the first marine *Paenarthrobacter* isolated at seafloor depth.

The GOM3 strain was recuperated from marine sediment obtained to a depth of 275 m in the Gulf of Mexico cultured with phenanthrene. Afterward, the strain was successfully isolated, but when its growth and degradation capacity in the presence of phenanthrene were tested, minimal growth was observed, and there were no significant changes in the consumption of hydrocarbons (Supplementary Figure 2). In a similar study performed by Wang et al. (2018), different strains belonging to species of the genera *Halomonas*, *Chromohalobacter*, *Thalassospira*, and *Alcanivorax* were isolated from a consortium enriched with phenanthrene, none of which could grow individually in the presence of this aromatic compound. These authors suggest that these strains probably play an important role in the lower pathways of PAH degradation since they found genes related to the degradation of central intermediaries in their genomes.

There are no experimental reports of *Paenarthrobacter* species that degrade benzoate, gentisate or protocatechuate. However, Meng et al. (2017) found genes related to the degradation of benzoate in the genome of *Paenarthrobacter nicotinovorans Hce-1* isolated from a polluted hyaluronic acid solution. In another study, Moraes et al. (2018) identified eight gene clusters related to aromatic degradation (including gentisate, protocatechuate, and catechol) in a novel *Paenarthrobacter* sp. HW13 strain isolated from a lignin-degrading consortium.

The benzoate degradation pathway has been commonly found in hydrocarbon-degrading marine bacteria in the Gulf of Mexico (Muriel-Millán et al., 2019; Rodríguez-Salazar et al., 2021). Raggi et al. (2020) reported the presence of recognized hydrocarbon-degrading bacteria and their metabolic potential through aerobic and anaerobic pathways in sediment samples collected from depths of 1,320, 2,966, and 3,010 m. The high diversity of Rieske oxygenases observed in the northern samples indicates potential for the degradation of diverse aromatic compounds, such as benzoate, benzene, toluene, phthalate, naphthalene, or biphenyl under aerobic conditions. Notably, Actinobacteria, in general, is not a dominant group in Gulf of Mexico sediments, and the *Paenarthrobacter* (or *Arthrobacter*) genus, in particular, is detected in very low abundance by 16S rRNA metaprofiling and is not part of the Gulf of Mexico taxonomic baseline (Godoy-Lozano et al., 2018).

Analyzing the genome of GOM3, we found five different dioxygenases, namely, 1,2-dioxygenase, catechol 1-dioxygenase, catechol 2,3-dioxygenase, protocatechuate 3,4-dioxygenase and gentisate 1,2-dioxygenase, involved in the activation of aromatic compounds. These enzymes are responsible for the GOM3 strain being able to grow in the presence of benzoate, gentisate and protocatechuate, with gentisate being the preferred substrate and exhibiting the highest volumetric consumption rate (Figures 3D, 4 and Supplementary Table 6). Genomic analysis revealed that this strain possesses four copies of the *genK* gene encoding the specific transporter gentisate. A remarkable observation within the genus is that all type strains use p-hydroxybenzoate as a carbon source but do not appear to assimilate the parent compound benzoic acid, suggesting that the activating enzyme benzoate 4-monooxygenase (EC: 1.14.14.92) is missing from these strains. However, the GOM3 strain possesses two genes encoding enzymes related to benzoate degradation *via* hydroxylation: a 4-hydroxybenzoate 3-monooxygenase [NAD(P)H] and two copies of 3-hydroxybenzoate 4-monooxygenase.

According to our experimental results, GOM3 has a substrate preference for gentisate, as this substrate has the highest consumption rate in every mix and when tested independently. However, the lack of a diauxic growth pattern in cultures with a mix of two or three carbon sources (Figure 4) suggests that the GOM3 strain could consume the three molecules at the same time, as seen in their simultaneous concentration decrease. The growth rate differences for each carbon source (gentisate, benzoate, and protocatechuate) could be attributed to several reasons. The possible greater gentisate influx could be due to the four copies of the *genK* transporter gene in contrast to one copy of the *benK* benzoate transporter and no copies of a specific (*pcaK*) protocatechuate transporter. A possible explanation for this redundancy in the gentisate pathway could be that this pathway produces fumarate and pyruvate. The former can be used to produce malate, and the latter can be used to produce oxaloacetate, feeding, and restarting the TCA cycle, as seen in *P. nicotinovorans* pAO1 (Mihăşan et al., 2021). The IclR regulator in the gentisate operon could regulate the glyoxylate shunt (Molina-Henares et al., 2006) to produce succinate before inhibition due to an increased malate concentration. On the other hand, the degradation of both benzoate and protocatechuate produces acetyl-CoA and succinyl-CoA. In this case, there is no direct access to the glyoxylate shunt, and the production of the other TCA cycle components could be slower, explaining the lag phase seen in benzoate and protocatechuate culture kinetics. The lack of a transporter for protocatechuate would suggest that its pathway catabolizes intracellular protocatechuate produced as an intermediary of other pathways.

Notably, we observed that protocatechuate is degraded by GOM3 more quickly in the presence of the other aromatic compounds. Some studies have found that in mixtures of aromatic compounds, synergic effects can be originated, such as the *Sagittula stellata* E-37 strain, which increased their growth rate in a mixture of benzoate/p-hydroxybenzoate (Gulvik and Buchan, 2013). In the case of gentisate degradation, we did not find the enzyme responsible for the subsequent reaction to transform 3-fumarylpyruvate (or acylpyruvate hydrolase and 3-fumarylpyruvate hydrolase); however, Pircher et al. (2011) found *in vitro* that a fumarylacetate hydrolase (FAH) also has acylpyruvate hydrolase activity, which could indicate that the enzyme belonging to the FAH family found in the gentisate pathway of *Paenarthrobacter* sp. GOM3 (Figure 3B) could convert fumarylpyruvate into fumarate and pyruvate. Further studies to understand the regulation of the related pathways in the GOM3 strain are required.

As part of the characterization of GOM3 as a new species, we compared the pathways involved in biogeochemical cycles of different *Arthrobacter* and *Paenarthrobacter* strains (Figure 2), resulting in very similar profiles across these strains. This result agrees with the sources of these bacteria, the majority of which were obtained from soil samples or sediment of natural springs. The analysis revealed an enrichment of key enzymes for nitrate reduction, ammonia assimilation and degradation of certain sulfur compounds. The nitrate reduction potential is consistent with metabolism under anaerobic or microaerophilic conditions (Jetten, 2008; Raggi et al., 2020), where nitrate is the second preferred electron acceptor after oxygen (Hensen et al., 2006). In particular, for the GOM3 strain, assimilatory nitrate reduction to ammonia or ammonification would consist of the internalization of nitrate/nitrite with MFS membrane transporters (*Nrt*), conversion of nitrate to nitrite by nitrate reductase (*nasA*) and conversion from nitrite to ammonia by the nitrite reductase (NADH) large subunit (*nirBD*). This ammonium is further converted to glutamine by glutamine synthetase (*glnA*) and is later incorporated in glutamate metabolism. In our analysis, there was no evidence of enzymes involved in nitrification reactions that convert ammonium back to nitrite/nitrate, as neither PFam was relevant for nitrogen fixation (Figure 2), indicating that the main source of nitrogen for the analyzed strains was environmental nitrate. Interestingly, the GOM3 and HW13 strains were the only strains with complete PFam domains involved in elemental sulfur reduction and tetrathionate reduction. Although the GOM3 and HW13 strains were isolated from different sources, namely, marine sediment and sugarcane plantation soil, respectively, both probably use H_2_ or organic substrates as electron donors to yield S^0^ and S_4_O_6_^–2^ as energy sources (Caspi et al., 2012). The GOM3 and HW13 strains were the only strains with 75% PFam completeness for dissimilatory sulfate reduction and oxidation, which is a predominant terminal pathway of organic matter mineralization in the anoxic seabed (Jørgensen et al., 2019).

In addition to these biochemical capabilities, we observed a unique battery of permeases in GOM3 that internalize a variety of substrates (Supplementary Table 1). The most prevalent permease was a peptide/nickel transport system with 14 related genes (KEGG orthology K02031-K02035) belonging to pathway ko02024 of quorum sensing (QS). Gram-positive bacteria use autoinduced peptides instead of N-acyl homoserine lactones (AHLs) as signaling molecules. Peptide autoinducers require specialized transport mechanisms to be excreted and imported back into the cell by a two-component system mechanism (Miller and Bassler, 2001). In species of the genus *Arthrobacter*, quorum-quenching activity in cross phylum interactions through the production of the depsipeptide arthroamide has been reported (Igarashi et al., 2015). In nonribosomal peptide synthetase systems, depsipeptide bonds are formed by nucleophilic attack on thioesterase-bound substrates (McClure et al., 2016). The prediction of biosynthetic gene clusters for secondary metabolites in GOM3 revealed the presence of regions for a nonribosomal peptide synthetase system, suggesting that this strain could have quorum-quenching activity. However, further analysis and experimental support are needed to corroborate this statement.

The experimental and genomic characterization of novel bacteria gives us the opportunity to explore the potential of hydrocarbon degradation that remains unexplored and that could be applied in bioremediation. Rational bacterial consortia design requires an understanding of their metabolic pathways and mechanisms that facilitate hydrocarbon degradation (i.e., chemotaxis, biofilm formation, biosurfactant production, and efflux pumps), as well as intercellular relationships (Xu et al., 2018). Individual draft genomes of hydrocarbon-degrading bacteria are key to the rational design of synthetic consortia with positive metabolic cooperativity. To achieve this goal, it is important to select not only the most efficient bacteria but also bacteria with different mechanisms for hydrocarbon degradation that can complement each other in a synthetic consortium.

In this way, we consider that *Paenarthrobacter* sp. GOM3 is a strain that can participate in a consortium with complex hydrocarbon-degrading strains with substrates, such as polyaromatics, resins, or asphaltenes (*Pseudomonas*, *Cycloclasticus*, and *Bacillus*) and with aliphatic degrading bacteria (*Alcanivorax*, *Rhodococcus*, and *Dietzia*) (Xu et al., 2018). There are no previous reports wherein this strain has been used in the bioremediation of contaminated sites; thus, exploring its biodegradation capacity is important.

As we are considering GOM3 as a promising strain for use in bioremediation, we explored its pathogenic potential using a lepidopteran model, *G. mellonella*, which is a good model for testing human pathogens (Hernandez et al., 2019). Our study shows that GOM3 reduced the larval health status in contrast with negative controls but not to the same level as the lower HI score for *P. aeruginosa* ATCC 27853. These differences could be related to the presence of virulence factors and pathogenic mechanisms, as was observed in experiments of extraintestinal pathogenic *E. coli* using larvae, wherein a relatively high virulence gene number killed larvae faster than a low virulence gene number (Tsai et al., 2016). Our calculated LD_50_ also supports this HI result. A higher number of CFUs of GOM3 are needed to kill half the population of larvae than of other pathogenic bacteria. To date, there are no LD_50_ reports of any opportunistic *Arthrobacter* pathogens. However, according to PATRIC annotation, the opportunistic pathogen *Arthrobacter* strain *A. woluwensis* DSM 10495 (Genome ID 156980.3) (84.21% ANI compared with GOM3) has the same *ilc* and *ilvD* virulence factors as our strain. If there is any interest in better understanding the virulence mechanisms of this strain, it will be necessary to perform future pathogenicity studies, such as establishing the activation of stress responses and repair mechanisms in larvae or applying murine model testing.

## Conclusion

*Paenarthrobacter* sp. GOM3 is a new species in the genus and is the first marine strain isolated on the seafloor, where its abundance is very low. There are few studies of the degradation of PAHs since the lower metabolic pathways are almost always ignored; however, the sequencing of its genome allowed the identification of enzymes that participate in the degradation of aromatic compounds. This is the first report experimentally demonstrating that a strain of this genus can grow in the presence of aromatic compounds, such as benzoate, protocatechuate, and gentisate, with the latter being the preferred carbon source. The GOM3 strain also has metabolic potential for nitrate reduction to ammonia, elemental sulfur reduction and tetrathionate reduction, as well as genes for QS. Additionally, we experimentally showed that the GOM3 strain can cause infection in the *G. mellonella* model, but it is less harmful than recognized pathogens and bears a wide range of antimicrobial resistances according to genomic analysis.

Due to the versatility in the metabolism of the GOM3 strain in the degradation of intermediate metabolites and the fact that it is not pathogenic, this strain could well be used in bioremediation as part of a degrading consortium in sites contaminated with aromatic hydrocarbons.

## Data Availability Statement

The datasets presented in this study can be found in online repositories. The names of the repository/repositories and accession number(s) can be found in the article/ Supplementary Material.

## Author Contributions

JR-D, AE-Z, AS-R, and LP-L contributed to the design and implementation of the research. JR-D, AE-Z, AS-R, and JR-V performed the computational and bioinformatic analyses. JR-D, LA, JR-V, and DC-A performed the experiments and analyzed the data. LP-L coordinated the group, managed the resources, and guaranteed their availability to perform the experiments and analysis. All authors contributed to the writing of the manuscript and approved the final version.

## Conflict of Interest

The authors declare that the research was conducted in the absence of any commercial or financial relationships that could be construed as a potential conflict of interest.

## Publisher’s Note

All claims expressed in this article are solely those of the authors and do not necessarily represent those of their affiliated organizations, or those of the publisher, the editors and the reviewers. Any product that may be evaluated in this article, or claim that may be made by its manufacturer, is not guaranteed or endorsed by the publisher.

## Supplementary Material

Supplementary Figure 1 *Paenarthrobacter* sp. GOM3 in *G. mellonella* infection model. **(A)** Health index (HI) scores of wax larvae over 5 days post infection with 100 UFC/10 μL. *P. aeruginosa ATCC 27853* exhibited an HI score of zero after 24 h (yellow line). The marine strain had a lower HI than the negative controls. **(B)** Dose-response curve with a range of *Paenarthrobacter* sp. GOM3 doses after 48 h of infection (*n* = 15). The gray area shows the confidence limits using the probit analysis with the “ecotox” library in R.

Supplementary Figure 2 Percentage of PAH degraded after 49 days by *Paenarthrobacter* sp. GOM3 growing in minimum medium with phenanthrene 0.02%. Assay made by duplicate.

Supplementary Table 1 Relevant functional annotations of unique genes detected in *Paenarthrobacter* sp. GOM3 by pangenomic analysis.

Supplementary Table 2 Genomic context of genes involved in the degradation of benzoate inside *Paenarthrobacter* sp. GOM3 genome and the percentage of identity of these genes with other strains.

Supplementary Table 3 Genomic context of genes involved in the degradation of gentisate inside *Paenarthrobacter* sp. GOM3 genome and the percentage of identity of these genes with other strains.

Supplementary Table 4 Genomic context of genes involved in the degradation of protocatechuates inside *Paenarthrobacter* sp. GOM3 genome and the percentage of identity of these genes with other strains.

Supplementary Table 5 Mean and standard deviation of kinetic parameters of *Paenarthrobacter* sp. GOM3 growing in different aromatic compounds individually.

Supplementary Table 6 Mean and standard deviation of kinetic parameters of *Paenarthrobacter* sp. growing in different mixtures of aromatic compounds.

Supplementary Table 7 Mean and standard deviation of final concentrations of each aromatic compound in abiotic controls.
