# Supplementary material for: Paenarthrobacter sp. GOM3 Is a Novel Marine Species With Monoaromatic Degradation Relevance
Source: Front Microbiol. 2021 Aug 3;12:713702. doi: 10.3389/fmicb.2021.713702 (PMC8369764; doi:10.3389/fmicb.2021.713702)
Supplement: Supplementary Figure 1 — Paenarthrobacter sp. GOM3 in G. mellonella infection model. (A) Health index (HI) scores of wax larvae over 5 days post infection with 100 UFC/10 μL. P. aeruginosa ATCC 27853 exhibited an HI score of zero after 24 h (yellow line). The marine strain had a lower HI than the negative controls. (B) Dose-response curve with a range of Paenarthrobacter sp. GOM3 doses after 48 h of infection (n = 15). The gray area shows the confidence limits using the probit analysis with the “ecotox” library in R. [file Data_Sheet_1.docx]

Supplementary Material


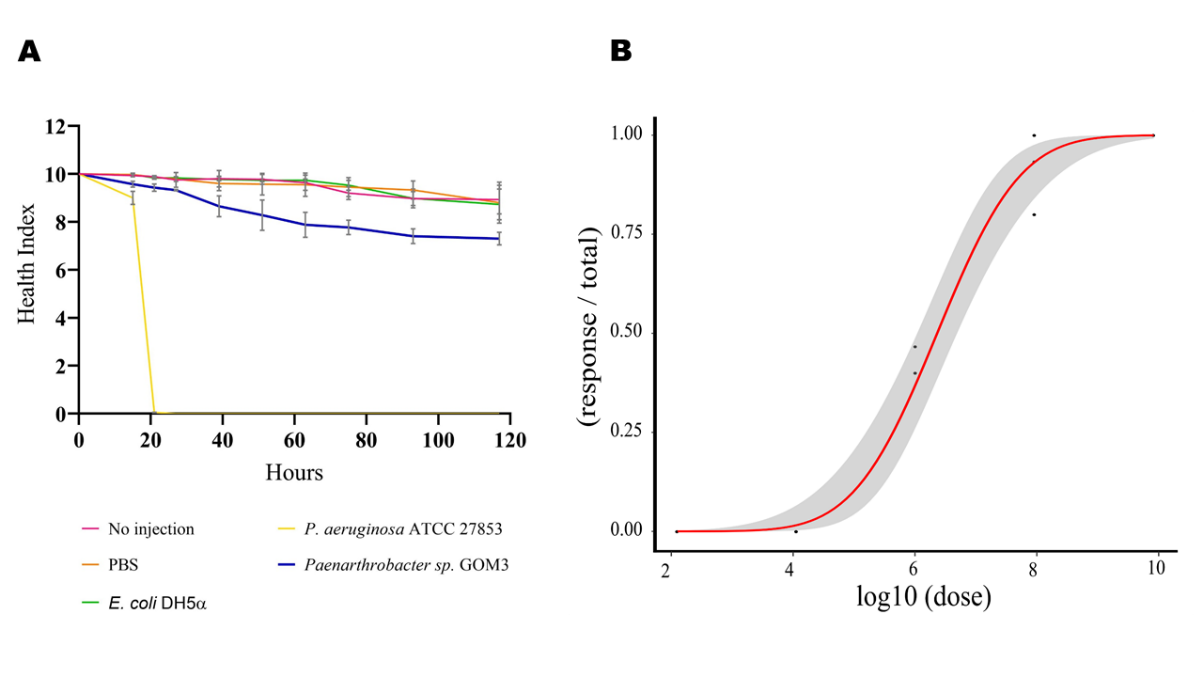


**Supplementary Figure 1.** *Paenarthrobacter sp.* GOM3 in *G. mellonella* infection model. A. Health index (HI) scores of wax larvae over 5 days post infection with 100 UFC/10 μL. *P. aeruginosa ATCC 27853* exhibited an HI score of zero after 24 h (yellow line). The marine strain had a lower HI than the negative controls. B. Dose-response curve with a range of *Paenarthrobacter* sp. GOM3 dosis after 48 h of infection (n=15). The gray area shows the confidence limits using the probit analysis with the “ecotox” library in R.


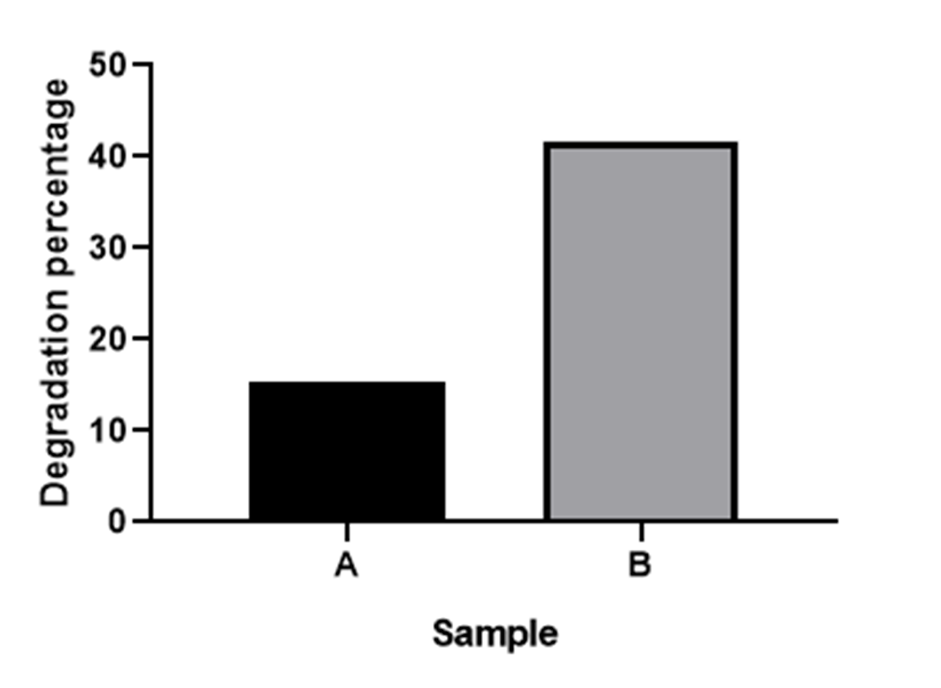


**Supplementary Figure S2.** Percentage of PAH degraded after 49 days by *Paenarthrobacter* sp. GOM3 growing in minimum medium with phenanthrene 0.02%. Assay made by duplicate.

**Supplementary Table 1.** Relevant functional annotations of unique genes detected in *Paenarthrobacter* sp. GOM3 by pangenomic analysis.
